# Expectations and Experiences Among Clinical Staff Regarding Implementation of Digital Pathology: A Qualitative Study at Two Departments of Pathology

**DOI:** 10.1007/s10278-024-01087-w

**Published:** 2024-03-28

**Authors:** Helene Koefoed-Nielsen, Kristian Kidholm, Marianne H. Frederiksen, Minne L. N. Mikkelsen

**Affiliations:** 1 CIMT - Centre for Innovative Medical Technology, Odense University Hospital, 5000 Odense, Denmark; 2 C*I2M - Centre for Integrative Innovation Management, University of Southern Denmark, 5230 Odense, Denmark; 3 Department of Pathology, Odense University Hospital, 5000 Odense, Denmark; 4 Department of Pathology, Hospital Sønderjylland, University Hospital of Southern Denmark, Aabenraa, Denmark

**Keywords:** Digital pathology, Implementation, Qualitative, Whole slide imaging (WSI), Management, Change management

## Abstract

The aim of this study was to assess and evaluate the individual expectations and experiences regarding the implementation of digital pathology (DIPA) among clinical staff in two of the pathology departments in the Region of Southern Denmark before and during the implementation in their department. Seventeen semi-structured interviews based upon McKinsey 7-S framework were held both prior to and during implementation with both managers and employees at the two pathology departments. The interviewees were pathologists, medical doctors in internship in pathology (interns), biomedical laboratory scientists (BLS), secretaries, and a project lead. Using deductive and inductive coding resulted in five overall themes and appertaining sub-themes. The findings pointed to an overall positive attitude towards DIPA from the beginning. The clinical staff perceived being rewarded already during implementation with benefits such as improved collaboration both inter- and intra-departmentally promoting better acceptance of DIPA. The clinical staff also experienced some challenges, e.g., increase in turnaround times, which affected and concerned staff on a personal level. Especially BLS expressed experiencing a demanding and stressful transition due to unexpected increase in workload as well as some barriers for a potentially better implementation process. The key findings of this study were a need for better preparation of staff through transparent communication of the upcoming challenges of the transition to DIPA, more system-specific training beforehand, more allocation of time and resources in the implementation process, and more focus on BLS’ work tasks in the requirement specifications.

**Supplementary Information:**

The online version contains supplementary material available at 10.1007/s10278-024-01087-w.

## Introduction

The implementation of digital pathology (DIPA) in the Region of Southern Denmark is a first-time achievement in Denmark and one of the first in the Nordic countries [1]. The clinical staffs of the Region’s pathology departments have been faced with a change in their workflow since the official start of the implementation at the end of 2020.

DIPA is an image-based environment that enables the acquisition, management, and interpretation of pathology information generated from a digitised glass slide [2]. In this way, the workflow for the pathologists changes from the traditional manual microscopy to visualising the tissue samples in a high-resolution image on a computer screen. Additionally, the biomedical laboratory scientists (BLS) now operate a scanner, perform quality control digitally, and distribute the tissue samples digitally to the pathologists. The preparation of tissue samples up until scanning remains the same [3, 4]. (See Appendix, Table 6 for full elaboration of changes in the workflow.)

DIPA has shown to be useful in the way pathologists can share tissue samples online and therefore consult with remote colleagues in the region or international specialists instantaneously [5]. DIPA is assumed to be the first step towards integrating artificial intelligence methods, algorithms, and computer-aided diagnostic techniques [6]. This will offer pathologists the tools to support the decision-making process in diagnostics and hopefully relieve them in terms of workload [1]. This is essential to achieve as tissue sampling is expected to increase in the future [1], due to an ageing population and growing referrals and the rise in initiatives to diagnose cancer earlier [6]. However, the number of pathologists is not expected to increase sufficiently to meet the growing demand [1, 7].

It is a strategic and political goal in Denmark to use digital technologies to improve the health sector [8]. It is therefore important to gain knowledge about how staff and management perceive the changes caused by increased digitalisation for other organisations in future implementations to better anticipate and handle the potential challenges.

As described above, implementing DIPA in a department entails a huge organisational change. The implementation of DIPA will have an impact on the daily workflow for numerous employees in the process of implementation. Furthermore, organisational change is considered a potential source of significant stress to employees [9].

Health researchers are increasingly appreciating and recognising the need for implementation science. Implementation science is a multidisciplinary research method created to increase the successful uptake of an intervention or new routine in, e.g., clinical departments, typically by addressing an underutilised practice. The aim of the field is to close the gap from research to practise by addressing the barriers or challenges that may stand in the way of a successful implementation [10]. Through social research, it aims to embrace broader than usual clinical research by including more levels than just the patient level. Levels like the provider team or group level and the organisational level are also thought to be potential barriers to implementation [11]. Implementation science suggests that simply measuring the productivity and effectiveness of implementations is insufficient. Researchers must recognise the need to evaluate not only endpoint outcomes [11], but also pay greater attention to audience and stakeholders’ perspectives as they are frequently overlooked, not acted upon, or reported [12].

Studies have examined the expectations, perceptions, and experiences of the staff towards the implementation of health technologies in a quantitative way [4, 13], but fewer studies in a qualitative way [7, 14] although it enables more in-depth assessment of opinions and attitudes towards, e.g., DIPA. This study is part of a larger ongoing mixed method study assessing the prerequisites and consequences of implementing DIPA [15]. This article will focus on qualitative data.

The aim of this qualitative study was to assess and evaluate personal expectations and experiences towards the implementation of DIPA among the clinical staff in two of the pathology departments in the Region of Southern Denmark. This was done through semi-structured interviews both prior to and during the implementation process of DIPA. The objective of this article is to highlight important facilitators, barriers, and potential benefits and challenges through the staff’s perspective. A second objective is to provide affected staff with sufficient insight into the process and what to expect. In this period of transition, capturing the perspectives of the clinical staff may contribute to a better understanding of the implementation process of digital technologies in health care. Hopefully, this study will help ensure an optimal change management in future implementations of DIPA.

## Materials and Methods

Data consists of semi-structured interviews with staff members from two pathology departments in the Region of Southern Denmark. The method used to gain better insight into the development in viewpoints is process evaluation [10] in which data is collected before, during, and after implementation from an observational viewpoint with no interference or feedback from the researcher to the implementation team.

This study will analyse interview data collected over two rounds. The first round was in the period from November 2020 to January 2021, prior to the implementation of DIPA. The second round was from October 2021 to March 2022, during the implementation of DIPA. Data from these rounds are partly used in the larger study [4]. Post-implementation data gathering will occur at a later stage.

### DIPA Solution

This regional project commenced with a pre-analytic and planning phase in 2016–2017. During this phase, the project group made international visits with other institutions that had transitioned to DIPA. The project group hosted a visit from an expert who provided insights into the implementation of DIPA in Leeds Teaching Hospitals [16]. The subsequent phase included preparation of requirements from the findings of several user groups, followed by bidding and negotiation rounds with potential system suppliers. For a complete overview of the implementation decision process, see [4] and Table 1.

The selected DIPA solution at the departments consisted of scanners and associated computers, as well as an image management system. The Hamamatsu NanoZoomer S360 Digital slide scanner (RRID:SCR_023761) is used for normal objects glass and Hamamatsu NanoZoomer S60 Digital slide scanner (RRID:SCR_022537) for macro glass. The software for the Hamamatsu scanners NZAcquire allows users to check the quality of the digital tissue samples. All glasses are scanned at × 40 except macro glasses which are scanned at × 20. When the glasses are scanned, they are released to an image management system by Sectra Danmark A/S. The image management system is contextually synchronised with a laboratory pathology system from the Danish company, Sirenia.

### Departments

Both pathology departments engage in academic and community practice. They differ in geographic location, size, and specialisation. Annually, the larger department handles just under 60,000 histology cases and employs approximately 45 pathologists, while the other processes around 20,000 histology cases and employs around 10 pathologists. Both departments largely consist of subspecialized pathologists, where the larger department covers more sub-specialised tissue samples. The departments had limited experience with digital pathology in terms of archive scanning only operating in × 20 and frozen section biopsy.

### Participants

To represent the entire department, the interviewed staff members consisted of representatives of BLS pathologists, interns, secretaries, and a project lead from the Region. The management was asked to select staff with various seniority across professions and different degrees of involvement in DIPA to join the study. The staff members and managers with time and interest were then interviewed.

### Data Collection

The 4th author completed 18 individual interviews prior to implementation in the first round and 18 interviews during the implementation of DIPA in the second round. The 4th author met with each interviewee at his/her workplace or online. Audio recordings of the interviews were captured by use of a Dictaphone.

### Interview Guide

The semi-structured interviews were based on an interview guide designed upon the McKinsey 7-S framework. This is a model designed to analyse the effectiveness of an organisational change consisting of seven internal elements that need to align for it to be successful. The designers of the model claim that a successful organisational change is affected by the relationship between Structure, Strategy, Systems, Style, Skills, Staff, and Shared Values [17]. The elements of the model can be divided into Hard S’s and Soft S’s, the first of which is considered easier to manage and change [18]. The McKinsey 7-S framework is suitable as a framework for investigating the implementation of DIPA due to its ability to highlight the internal conditions of the organisation. Based on this model, five themes were created for the interview guide (Table 1).

**Table 1:** Interview guide structure with McKinsey 7-S framework

**McKinsey 7-S elements**	**Themes**	**Elaborated in relation to DIPA**
Strategy	The perspective of DIPA	The general purpose of DIPA perceived by the staff and the management. Their initial expectations, concerns, and excitement
Style	The implementation process	The management style used to implement DIPA by means of resources, communication, and success criteria. Furthermore, focus on the transition, barriers, and facilitators of the implementation process
Staff and Skills	Readiness for working with DIPA	Human resources and talent management in connection to the process. Capabilities and competences through training and readiness of the staff and management enabling them to realise DIPA
Systems and Structure	Everyday working with DIPA	The arrangement of workstations and procedures and the impact on the daily workflow through changes, benefits, and challenges of DIPA
Shared Values	The motivation for pathology	The common values and culture of the organisation including the motivational factors of working in pathology and the acceptance and importance of DIPA for the department

To investigate the different perspectives from the management and the staff, respectively, the interview guide was parted in two. The themes remained the same, but the questions were designed to fit according to the perspectives of the different groups. In the second round, the questions were adjusted to fit the changes during the implementation of DIPA (see Appendix, Tables 7–10 for the entire interview guide).

The interviewees were allowed to diverge from the questions and occasionally asked elaborating or clarifying questions to better portray the opinion of the interviewees.

### Data Analysis

The 4th author transcribed the first round of interviews using the verbatim method for further analysis. This researcher transcribed the second round of interviews likewise. The interview data was stored in the secure database OPEN at Odense University Hospital [19].

Data analysis was performed using the NVivo version 14.23.2 (RRID:SCR_014802)—qualitative data analysis software. Coding was initially performed manually using a deductive approach following the structure of the interview guide. First, it was coded into the five themes and afterwards sub-coded into sub-themes. The sub-themes were then inductively coded according to the themes emerging within the answers. Expectations and experiences were then condensed, summarised, and compared within each theme and presented in the results. If an overall tendency or opinion arose around a particular theme, it is presented in the article. In the presence of a particularly relevant quote from an interviewee, it is incorporated to best emphasise and represent the common perception. The quotes were translated from Danish to English in the best way possible to preserve the attitude, character, and opinion of the quote. Participants were offered to review their translated quotes during a round of consent to publish with the possibility to rephrase their quotes. The validity of data was hereby ensured. Only a few participants rephrased their quotes. These quotes were evaluated by the 1st author and accepted due to retained expression.

## Results

**Fig. 1:**
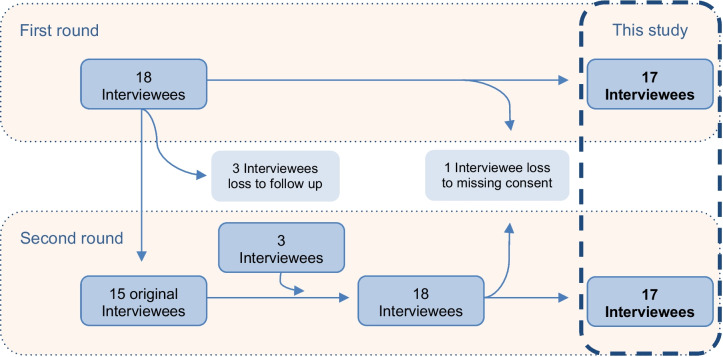
Study flow chart of who participated in this study over the first and second round of interviews. First round of interviews consisted of 18 interviewees. Three interviewees were lost to follow-up for the second round leaving 15 original interviewees to participate in the second round of interviews. Three new interviewees were enrolled making up for the loss, again totaling 18 interviewees in the second round. While obtaining consent to this study, one original interviewee who participated in both rounds was omitted due to missing consent. Therefore, this study contains 17 interviewees from the first round and 17 interviewees from the second round

In both rounds, prior to and during implementation, 18 interviews were conducted. In the second round, 14 of the original interviewees participated again, while three who were lost to follow-up were replaced with three new interviewees. One original interviewee did not respond to consent obtained for this investigation and data therefore consists of 17 interviews in each round (Fig. 1).

### Interviewee Characteristics

Out of the overall 20 interviewees, the majority were women, about two-thirds worked at the largest department. Doctors and BLS were equally represented by profession (Table 2).

**Table 2:** Sample characteristics of the interviewees (*N* = 20)

Interviewees	*N* (%)	Gender		Years of experience in pathology				Size of department	
		Female	Male	0–2	3–5	6–9	> 10	Large	Small
Consultant	4 (20%)	2	2	0	0	2	2	3	1
Senior registrar	2 (10%)	2	0	0	0	1	1	1	1
Interns	2 (10%)	1	1	2	0	0	0	2	0
BLS*	7 (35%)	4	3	1	4	1	1	5	2
BLSmanager	1 (5%)	1	0	0	0	1	0	0	1
Secretary	3 (15%)	3	0	0	1	0	2	1	2
Project lead	1 (5%)	1	0	0	0	0	0	0	0
Total	20 (100%)	14 (70%)	6 (30%)	3 (15%)	5 (25%)	5 (25%)	6 (30%)	12 (60%)	7 (35%)

Project lead is only presented in Gender therefore total ≠ ∑100% in the other categories

*BLS biomedical laboratory scientists

### Expectations Before Implementation

Thoughts on the general purpose of DIPA raised many expectations before implementation according to the staff and the management. The staff described expecting to get increased cooperation between the four departments in the Region of Southern Denmark and potentially all of Denmark or maybe even by time with peers worldwide. They described hoping that DIPA will make it possible to sustain smaller remote departments by relieving and sharing tasks, enabled by the geographic flexibility of digital solutions.

The interviewees expressed prior to the implementation that they were convinced that DIPA would play a vital role in progressing pathology into the future and setting the right direction for the department. The staff described believing that implementing DIPA perhaps will help maintain a higher standard and specialisation of diagnostics with easier and faster consultation. Most staff mentioned artificial intelligence being one of the purposes and goals for implementing DIPA. Nevertheless, they also noted that achieving this goal will take some time, due to the ongoing development needed to effectively integrate AI into the clinical workflow. The staff also expressed the hope of a more modern system attracting younger generations of pathologists.

The excitement in the department was tangible and attitudes towards DIPA were positive. The staff expressed awareness of a potential challenging time ahead, but remained optimistic: *Of Course, it will be hard in the beginning…. And it will be a challenge, but I am sure we will get through it (Intern).* The staff was able to see the future possibilities but had realistic expectations about what DIPA would provide and that it would take some time to get acquainted to and maximise the potential yield.

#### Concerns

Some concerns expressed by staff prior to implementation encompassed technical problems, including trust issues with digital solutions and patient safety. Other concerns were centred around their work situation including task changing, possible outsourcing, and especially the secretaries worried if they would still have a function in the future and had trouble seeing the benefits for their profession.

The staff described themselves as passionate about their work and highly aware of the impact it has on patients: *You take pride in what you do, so the patient gets a quick and correct answer (BLS).* When asked prior to implementation of DIPA, most employees did not assume DIPA would have much influence on what is important for them individually. However, several BLS expressed that they highly value their craftsmanship and worry that the level of craftsmanship will diminish in the future. One BLS answered this could make him identify less as a BLS in the future due to all new technology, whereas the pathologists in general did not think of the microscope as an identity marker.

Prior to implementation, the staff and the management expressed concerns about scepticism from the pathologists towards DIPA and their potential opposition to its implementation. Some of the pathologists wondered about the prematurity and the necessity of DIPA being implemented: *The images are really good with high quality… But it is not better than the microscope, and there isn’t any indication in our system or DIPA which provides decision support. Therefore, it just becomes an add-on to the normal diagnostics (Consultant).*

### Experiences Regarding Implementation

#### Communication

In general, the staff expressed that they found the transition period challenging, stressful, and chaotic due to a lack of information and proper notice. Some BLS and interns described feeling as though they did not receive enough information, which created uncertainty. They expressed a lack of transparency, as if only a part of the information was carried on from the consultants and senior registrars. *I understand that it has been a stressful task for the management to set this in motion. But there really has been a lack of communication. I believe there have been a lot of frustrating incidents (BLS).* However, most of the consultants and senior registrars describe feeling sufficiently informed.

One of the senior registrars, who coincidentally alternated departments during the transition period, described thoughts on the communication. The senior registrar’s impression was that there was a surplus of information in the smaller department where everyone was involved in everything, whereas the information did not travel as far in the larger department. However, some staff mentioned that everyone received emails with updates, but these were too long, too broadly encompassing, and not read due to lack of time.

#### Training

The staff described not feeling sufficiently prepared prior to DIPA and described it as being faced with unknown territory. Likewise, they expressed a lack of information of expected challenges to improve preparedness as well as not receiving enough training prior to DIPA. The pathologists particularly expressed the need for follow-up training or *…at least simultaneously (…) it is annoying when you discover some kind of feature that could have saved time, which you find out half a year later (Senior registrar)*. Furthermore, they expressed interest in getting to know more tools in the system but found it difficult to allocate the time to explore on their own.

Instructions for the BLS took place as “over-the-shoulder”-training which they expressed liking but they described the need for more resources and time allocated to it. Furthermore, the BLS were unsatisfied with the sparse amount of instructions in the quality control since this became a major work task change.

Both BLS and the pathologists confirmed that help was always available from other colleagues or super-users. The staff praised the super-users for their tenacity but wished on their behalf that they had received more training beforehand and had gotten allocated time to help. *It has been challenging. (…) I don’t think I had the tools I needed to establish it really well, or to at least train the work team and to train my colleagues and such at home, because among other things we didn’t gain access to the testing system (BLS super-user)*. The missing test system, originally planned to be installed before implementation, is something many mention as a shame not getting to try out beforehand. The staff thought it would have clarified things and perhaps helped calm the nerves if they could have experienced the system in real life.

Overall, the management echoed the frustration over the absence of the test system and stressed the importance of being able to demonstrate and trial it with staff beforehand. Management attributed some of the deficiency in training to the suboptimal conditions resulting from the COVID-19 situation. However, they conveyed appreciation for the efforts of the dedicated super-users and their valuable contributions to the training.

#### Workflow/Load

During the transition phase to DIPA, the pathologists were involved in DIPA incrementally. According to the staff, this affected the BLS’ workflow of distributing tissue samples negatively because now there were two groups of pathologists: some working digitally and others still analogously. The BLS described their workflow during this period as being time-consuming and complicated, as it was their responsibility to keep track of which pathologist had transitioned and who had not: … it was *curse* annoying to put it lightly, but I understand that it takes time for the pathologists to get used to it (BLS).

The pathologists, on the other hand, found this period to be relatively problem-free and smooth, mostly complaining about the DIPA system not working smoothly together with the existing pathology system.

The pathologists expressed how the change in workflows due to DIPA led to the workload being the same or less than before. The BLS described a greater workload, while the secretaries experienced less. The management described the same perception as the staff: *I think the pathologists are more positive, because they were rewarded with something, whilst the BLS have just gotten more work to do (Project lead)*.

Furthermore, the management struggled with allocating enough resources for the partially increased workload caused by the DIPA implementation. Management expressed being surprised by how demanding it was for the BLS and how hiring more BLS at that time was unfortunately difficult due to the high recruitment of BLS’ in the COVID-19 effort.

#### Management/Strategy

The management described that they had not pre-emptively imposed any particular theory of implementation for DIPA. A rapid implementation was mentioned as a high priority, bearing in mind not to overburden the staff. The management explained how they were hesitant to set up the intermediary milestones for implementation success criteria. According to the management, the implementation approach revolved around what was technically possible from the laboratory’s point of view*.* Furthermore, the management expressed that they consciously involved the BLS: *It’s been important for me that it was a joint project between BLS and pathologists (…) and that has proven to be wise, because it’s been a large task and something new for the BLS to become acquainted with, and I believe they’ve made it less complicated because they feel that they’ve had an influence on the process (Consultant)*. A consultant in the management underlined the importance of the management also working with DIPA during the implementation process to understand the challenges on the same terms as the employees. He further explained the importance of it not being an outside academic trying to lead the department, but rather one from the department.

It was mentioned repeatedly by the management how important it was for them to give time for the staff to adapt to it and be sure to diagnose the same digitally as analogously. The management described being adamant about the way forward, without exceptions: *We must be digital and that might take some people longer than others, but the result must be the same (Consultant)*. This was verified by the staff: *The management has persistently backed up that this is what we’re going to do (…) they’ve been good at ensuring that things didn’t decelerate (…) so that everyone is on board, and we don’t miss anything (BLS)*.

#### Barriers and Facilitators

The staff and the management were also asked directly to identify barriers and facilitators that made this implementation process more or less optimal (see Table 3). The most highlighted facilitator was key persons who were very dedicated to making things work. Another facilitator was the positive reception by pathologists in contrast to the BLS group that experienced an increased workload and frustration working in a system not optimised for their use case, causing low motivation and excitement, thus categorised as a barrier. Staff also generally expressed that the implementation was aided by inter-regional meetings and sharing experiences midway. The pathologists were happy with the optimal image viewing system and its high quality. A specific barrier mentioned for a smooth implementation in one of the departments was the simultaneous department relocation that caused DIPA to be deprioritised.

**Table 3:** Facilitators and Barriers would benefit from being horizontally centered

**Facilitators**	
**Key persons** *Some people have been very dedicated, if it hadn’t been for them, we wouldn’t have been here today. (Project lead)* **Positive approach from pathologists** *The fact that the pathologists have been so optimistic that there has been little to no resistance on the pathologists’ side, and they have accepted it so well. (BLSmanager)*	**Functioning system for pathologists** *That it actually works—the images have been quite good (…) as well as the good monitor and a fast system to use. (…) because when they’ve [tissue samples] actually been scanned (…) and reported ready from the laboratory, it has worked very well. (Consultant)* **Inter-regional cooperation** *… We have meetings where we talk internally, but also across hospitals (…) having regular meetings where you exchange experiences all the time. That’s definitely the way forward. (BLS)*

**Barriers**	
**Relocation of department simultaneously** *It was a lot to ask of the BLS and the pathologists to move and install a new department while also introducing a brand new diagnostic setup with DIPA. I believe that’s been a bit stressful. (Consultant)* **System not compatible for BLS** *… That there might have been a system, which would have been better to implement (…) One, which would have been more BLS friendly. (Consultant)*	**Slightly increased opposition from BLS** *At the time it occurred to them [the BLS] that it would be more resource demanding than expected (…) There has been some resistance from their side (…) when introducing new technology, we would like to be relieved and not burdened. (…) There will be years now where it is more of a burden for the BLS, and where there is no real gain for the pathologists, but hopefully we are awaiting a future where it will be easier for the pathologists. (BLSmanager)*

### Experiences with DIPA

Asked about the importance of DIPA for the department, a common belief amongst the staff was that they were now recognised and acknowledged as a more modern workplace. They were proud to be trendsetting, more high tech, and to be the first to implement DIPA in Denmark.

In the current phase of implementation, the staff already had experienced successes in achieving DIPA-related benefits. This included the benefits of having digital tissue samples enabling more rapid sharing of images with colleagues and even remotely located colleagues. This feature was greatly appreciated by the pathologists, expressing excitement to increased levels of cooperation with other departments. This excitement was also shared by the BLS group that further added topics such as remote consultation, easier coverage during holidays, improved survivability for smaller departments due to more accessible remote expertise, and better supervision for interns to the list of benefits that had already had an impact on both departments. Other benefits frequently mentioned by pathologists were the addition of software tools and applications in the digital system, the ability to work from home, and improved ergonomy. (See Table 4 for additional identified benefits and elaborating quotes by staff.)

**Table 4:** Benefits of DIPA identified by the staff and the management

**Benefits**	Representative statements
**Digital tissue samples**	
Distribution	BLS: *We used to spend a lot of time on distribution and that part has become less, like really minimised (…) it was really difficult sometimes and it’s a lot easier when it’s all on the screen.* BLS: *They (The machines) are just effective and don’t make the same mistakes as we do.*
Digital archive	Consultant:* If it’s digitalised, you can look at them straight away (…) Then you would do it more often.* Secretary: *It’s a benefit that we can send the glasses digitally, and we don’t need to find them physically.*
Quality control	Consultant: *The digitalisation has impacted the lab with regards to how the quality control of tissue slides is performed. A new standard for an "optimised" slide has been set—as a good scanning result is now essential.*
**Sharing images**	
Consultations abroad	Senior registrar: *Sharing images and consultations, consulting with your colleagues in the house or outside the house, is very good. It gives very good opportunities.* BLSmanager: *As soon as the BLS are finished in the laboratory, then they [pathologists located differently] can just download them equally as if there was a pathologist in the office at this unit and respond to it.*
Cooperation	BLSmanager: *The department has a higher chance of being sustained now (…) Because we can get help faster and easier from some of the larger units, where there might be more expertise in some fields or when it comes to holidays or sickness, where we are very vulnerable.* BLS: *The fact that we helped each other in the summer holidays, I think of as a major advantage (…) I have no doubt it will be used for other holidays or even sickness.*
Supervision	Consultant: *There will be better supervision. There are also multiple who can view simultaneously.* BLSmanager: *They think it’s really nice, that it’s a tool education wise, that you can look at the screen (…) they said it was easier to point: It’s this cell we are talking about.*
**Pathologists’ digital workflow**	
Applications on the system	Consultant: *You can look at multiple tissue samples at the same time.* Intern: *It is a huge benefit, when you have to measure the thickness of a melanoma, that you can just drag a line, and then you know it’s calibrated. Then you don’t have to do all sorts of stuff with a ruler.* Senior registrar: *One thing really smart is you can always see if you have overlooked the entire glass, so you would know if you skipped a segment.*
Work from home	Senior registrar: *You can simply do work from home. It gives greater flexibility in your everyday life.* Consultant: *It allows for more flexibility, e.g. to take your child to a doctor’s appointment at 2 p.m. and then put in a couple of hours from 5–7 p.m- or whatever fits with dinner. Working from home allows you to move the samples one step further along, thus reducing delays in starting additional lab analyses, meaning they can be ready sooner. This allows for faster pathology reports.*
Ergonomics	Consultant: *Most of our staff feel that the ergonomics have been improved by being less dependent on the microscope. For me personally, the difference is not so great, but we all enjoy the new possibilities which put less strain on the neck, back and arms/hands.* Consultant:* Previously you were physically locked to the microscope (…) You can keep up with the weather outside, and if someone enters the room (…) You are more present in the room (…) The operating position is more comfortable.*

When asked about DIPA-related disadvantages, the staff and the management expressed a preference to refer to them as challenges. The challenge that affected the staff the most was the increased turnaround times. The staff attributed this feeling of frustration to the fact that they already had an effective and optimally functioning laboratory, so it was going to be hard to improve. The BLS also expressed having problems keeping up due to scanner and server capacity leading the management to introduce shifted work schedules to increase the active scanner time. Another frequently expressed challenge, mostly mentioned by BLS, was the absence of an established method to assess results of quality control of the scanned tissue samples. This imposed on the BLS’ need to subjectively evaluate the quality control results, generating feelings of uncertainty. A prominent challenge apparent in the interviews with pathologists was their altered workflows and what those alterations brought about. These are challenges such as personal uncertainties regarding proper handling of digital tissue samples compared to working with them physically which they were used to, being more in touch with the process. Other challenges expressed by pathologists included the inconvenient switch between operating two different systems and the loss of the analogue fine adjustment in the microscope. Additionally, they mentioned the possibility that there might arise an expectation of logging on outside regular working hours in the future caused by the combination of increased remote accessibility and shifts in response time patterns. (See Table 5 for additional identified challenges and elaborating quotes by staff.)

**Table 5:** Challenges with DIPA identified by the staff and the management

Challenges	Representative statements
**Increased turnaround times**	
Response time delay	Consultant: *… It’s slower and we can’t comply with our response times. (…) when you introduce extra steps, then someone else must work faster (…) we have had a super effective laboratory, which is hard to beat.* BLSmanager: *It’s still a challenge with samples with three days’ response time, because the scanning process makes us dependent on whether there are extra orders, then we are a half to an entire day late getting the samples to the doctors.*
Scanner capacity	BLSmanager: *Theoretically we are not under-dimensioned with scanners (…) The problem is that we don’t feed the scanner an equal amount every hour during the day, like the theory is based on.* Consultant: *Each one of our scanners can hold a total of 360 slides. However, scanning the full load would take approximately 10 h, thus locking up capacity for a long period. We realised that continuous loading and running smaller loads per scanning cycle freed up capacity and allowed for flexibility and quick scanning of samples that need to be reviewed urgently by the pathologists.* BLS: We have to work longer because of those *curse word* scanners, and that’s just another disadvantage, because it takes longer. [The management introducing shifted work schedules to increase active scanner time]
Server capacity	BLS: *The entire region runs on the same servers. So, there are certain times of the day where you know: Now it runs slowly (…) and then it could be two hours until it is uploaded.*
**Quality control**	
Lacking guidelines	BLS: *It is very subjective how people review the tissue samples…*
Higher standard	BLS: *We can now see them (tissue samples) microscopically, whereas before we only saw them macroscopically when we distributed them (…) so it’s no longer permissible to submit something ugly or useless.*
Insecurity	BLS: *When is it good enough? Because you can’t every single time be like: This must be 100% tip-top (…) So it’s the tempo, relative to what you really want to do, I must get used to.*
Case-based system	Consultant: *It is a huge barrier that it is a case-based program for them.* [The BLS can’t sort the tissue samples according to staining etc.]
**Pathologists’ digital workflow**	
Lack of physical glass	Senior registrar: *I must be a lot more careful than previously (…) with the same patient (…) because the sample might be the correct number, but then it might not be the sample you are looking at (…) In this way it is relatively risky because you can’t keep track in the same way.* Consultant: *You have to in some kind of way make your own system to remember which you have to follow up on, and you once had the physical glasses as a reminder in a pile.*
Images out of focus	Consultant: *There are of course some things you have to get used to with the depth in the images, which you can’t adjust, if the tissue sample isn’t good (….), because you can’t use the fine adjustment.*
Workflow	Consultant: *The feeling of not knowing when you will receive them [the tissue samples] (…) Your day has become less predictable (…) We are also not able to schedule our day.*
Accessibility	Consultant:* Now we more often work till later in the afternoon, because they [the tissue samples] aren’t ready until then. (…) The problem is that we also come in early to order immunes.* Consultant: *The flip side of the coin is that working hours are now less strictly defined. It can be stressful to be able to check on the progress of your assigned samples from home and perhaps keep working until late. Some will manage this better than others.*
Less contact with colleagues	Consultant: *The collegial relationship between pathologists and BLS has deteriorated (…) not because we aren’t friends, but simply because you no longer talk with each other. You don’t need to go there [to the laboratory], so we work more isolated, I think.*
Cooperation between systems	Intern: *I don’t have a good overview of my samples (…) if there weren’t two systems* [pathology system and DIPA]*, I think I would be much better able to keep track.* Intern: *It would have been smarter if it was all in one system (…) It’s a bit annoying having some functions in one of them and some in the other.*
**Secretaries’ tasks**	
Reactions from clinics	Secretary: *When our samples were delayed, we received really many inquiries, on mail, on telephone, and some even came here and asked. So that was a very harsh period, with a lot of unpleasant conversations.*
Consulting outside the region	Secretary: [Sending outside the region] *has given us some challenges. It would be smart if everyone had the same system.*

## Discussion

Results from this study indicate that the staff at two pathology departments in the Region of Southern Denmark generally had high expectations towards DIPA. Both BLS and pathologists had similar preconceptions of DIPA, expressing interest, excitement, and general positivity of advancements in the field of pathology. The staff also had some concerns regarding the shift in workflow and introducing a new technology.

During implementation, the staff has experienced some benefits of DIPA but also identified some challenges. Lack of communication, resources, training, and a clearly formulated strategy were among experienced challenges. These caused feelings of distress, uncertainty, and insufficient involvement. Despite all this, the current acceptance and persistent positive attitude towards DIPA appear to be dependent on the assumption that the challenges will be solved going forward.

When discussing the perception of the implementation process of DIPA, it is important to keep in mind that these emotions are expressed *during* the implementation phase. Therefore, frustrations may appear particularly heightened, but it does not imply that these challenges were not addressed or managed. It is merely a momentary snapshot amidst the intense transition.

The research findings of this study indicate that several of the expectations regarding DIPA were indeed met, such as enhanced collaboration with other departments or colleagues. However, notable disparities between anticipated outcomes and actual experiences were encountered, particularly concerning turnaround times. Despite initial expectations of expedited processes, challenges arose during implementation, resulting in difficulties in achieving even baseline turnaround times. This disjunction between expectations and realities emerged as a significant personal concern among staff members.

Regarding contrasting expectations and experience outcomes, an observation emerged. Initial apprehensions centred around potential resistance from pathologists towards the new system. Surprisingly, pathologists exhibited a predominantly positive stance. Conversely, the BLS who encountered a pronounced alteration in their operational procedures and workload experienced a slightly different perception towards DIPA from the original excitement. Perhaps this will change when DIPA becomes more established in the laboratory as experienced in other labs [20].

By use of quantitative analysis, a previous study prior to implementation [4] found overall high expectations, motivation, and readiness for upcoming changes in the pathology departments as well as *the perception of* no huge barriers ahead. In contrast to these findings, the actual *experienced* barriers may have caused a more negative attitude during the implementation process. This might have been avoided if the appropriate barriers could have been identified and addressed prior to implementation. The specific barriers may not be universally relevant for other implementations, except exemplifying the influence unexpected barriers can have on the staff.

The findings of this article match multiple findings in other implementation science studies. First to mention is the importance of the individual and how an implementation process affects them. *Individuals are carriers of cultural, organisational, professional, and individual mindsets, norms, interest, and affiliations [12, page 5]*. This study found that the staff felt greatly impacted by being temporarily obstructed in providing the service they normally practise, affecting a core pride and representing a motivational factor for the staff, which was fast turnaround times and good service for the patients. This was the largest and most frequent basis for the staff’s frustrations and concerns. It is out of the scope of this study to comment on factual turnaround times, but it matches findings in other studies showing worsened turnaround times [21, 22]. However, it is noteworthy that other studies have indicated departments achieving enhanced turnaround times as digital workflows become more established, offering a hopeful prospect for staff members to anticipate and strive towards [23].

Another match with implementation science research is the importance of adaptation of interventions. *Without adaptation, interventions usually come to setting as a poor fit, resisted by individuals who will be affected by the intervention *[11]. The results of this study indicate a missed realisation of the importance and focus on the BLS’ work tasks, when designing the requirement specifications. This might be due to the management taking inspiration from DIPA implementations from other countries, specifically the task of quality assurance, a task performed by BLS in Denmark, unlike other countries. This poor adaptation of DIPA resulted in the BLS experiencing a more stressful and challenging transition.

Lastly, this study also found communication to be crucial for a successful implementation, resembling other findings in implementation science. *The importance of communication is clear. Making staff feel welcome, open feedback and review among peers and across hierarchical levels, clear communication of mission and goals.. to contribute to effective implementation [11, page 8]*. The interns and BLS felt a lack of transparency and communication, unlike consultants and senior registrars. This perhaps reinforces the staff’s feeling of information being shared uncoordinatedly across professions and hierarchy, contributing to a sense of exclusion. According to the staff, the management failed to communicate the expected challenges related to DIPA. This is perhaps caused by the unconscious communication of exclusively positive aspects of DIPA originating from the management in an attempt to promote readiness and morale towards DIPA among the staff. The consequence is that lack of communication, unintentional or not, induces misrepresentation. To avoid misrepresentation in future DIPA implementations, the management should give a complete, realistic, and authentic explanation of potentially positive and negative outcomes to reduce the uncertainty of the staff [24].

Using the McKinsey 7-S framework as a guideline for investigating expectations and experiences among the different stakeholders in the pathology departments made it possible to understand the influence that changes caused by the implementation of DIPA had on the staff. McKinsey 7-S framework claims that changes in one element affect the other elements and cause instability, as is confirmed in this study. The changes in hard elements *Systems* and *Structure* fx workflow and workload have substantially affected soft elements such as *Staff* and *Shared Values* fx integrity, motivation, and pride. To restore balance in the organisation, the department ought to make the adjustments necessary to ensure a better alignment of the 7-S elements. Failing to do so might induce difficulties for staff to appreciate the purpose of DIPA and change perceptions of it.

A limitation of this study using the qualitative method is that the statements are not supported by factual data confirming the correctness. The McKinsey 7-S model also does not encompass organisational effectiveness or performance explicitly, but this is being investigated concurrently in the Region of Southern Denmark and will be analysed and presented in a further study. Another limitation is that selection bias cannot be ruled out because the department managers selected the interviewees. However, it seems the responses were not unilaterally positive or negative only. Furthermore, too few secretaries participated, making it difficult to conclude much on their behalf. Further research on their role in DIPA is needed.

**Fig. 2:**
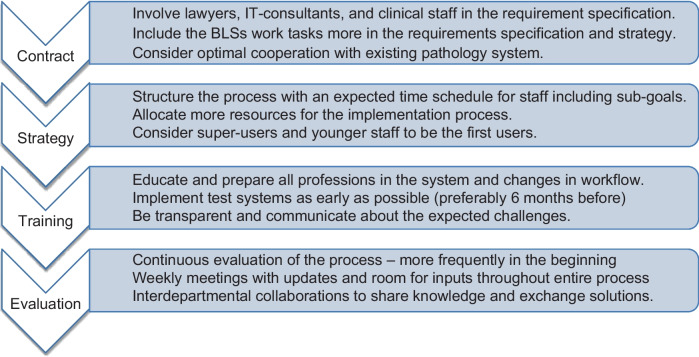
Take-home messages extracted from interviewees’ statements from the second round of interviews

In return, examining the reception of DIPA from a qualitative point of view enabled exploring the implementation process in greater depth and from different perspectives. It induced concrete statements that can be summarised into focus points to keep in mind for other subsequent implementations (Fig. 2). The results identify not only what can be done differently in another implementation of DIPA but also identify the challenges of what needs to be improved in this current implementation. One such consideration is the discovery of the importance of engaging all professions, including the crucial contribution and dependence of the BLS in DIPA.

The findings can provide insight into what the staff and the management can expect when facing an implementation process of DIPA. It should be a priority in an implementation process such as this one, to research the barriers, challenges, shifts in workload, expectation, and feelings identified by other studies. Even if the hurdles are unavoidable, informing and reconciling expectations of the staff may improve the implementation experience.

Using process evaluation provided important insight into expectations prior to implementation to investigate development in attitudes, even though most of the results in this study are derived from data collected during implementation. Therefore, process evaluation is arguably more useful when data will be collected post-implementation in a future study and compared with the findings of this study.

In the future study, data will be collected when DIPA has become routine. The aim of that study will be to investigate whether the perceptions of DIPA have changed and if the challenges identified hitherto have been addressed, new issues emerged, or if more benefits have been realised.

## Conclusions

In conclusion, clinical staffs from the two pathology departments in the Region of Southern Denmark were overall found to be optimistic and excited towards transitioning to DIPA. From their point of view, the implementation process was suboptimal. The results in this paper highlight pivotal topics to be addressed. Although still in the implementation phase, the staff already appreciates some DIPA-related benefits, such as the ability to work from home, easier distribution of tissue samples, collaboration inter- and intra-departmentally, and the capability to sustain smaller remote departments.

Based on the findings, it seems to be important to prepare staff through communication of the upcoming challenges of the transition to DIPA, more system-specific training beforehand, more allocation of time and resources in the implementation process and more focus on BLS’ work tasks in the requirement specifications.

Achieving this level of insight into how the implementation of DIPA affects the staff provides important knowledge about the relevance of focusing on change management during implementation of digital health solutions. The findings of this study may inspire staff or managers prior to implementing DIPA in their departments to ensure preparedness and a good transformation.

## Supplementary Information

Below is the link to the electronic supplementary material.

Supplementary file1 (DOCX 33 KB)

## Data Availability

The transcripts of the interviews are not publicly available because of the rather small sample size from identified departments, and therefore, the anonymity of the participants is not secured.
