# Supplementary material for: Expectations and Experiences Among Clinical Staff Regarding Implementation of Digital Pathology: A Qualitative Study at Two Departments of Pathology
Source: J Imaging Inform Med. 2024 Mar 28;37(5):2500–12. doi: 10.1007/s10278-024-01087-w (PMC11522200; doi:10.1007/s10278-024-01087-w)
Supplement: Supplementary file 1 — Supplementary file1 (DOCX 33 KB) [file 10278_2024_1087_MOESM1_ESM.docx]

**Journal of digital imaging**

Expectations and experiences among clinical staff regarding implementation of digital pathology: A qualitative study at two departments of pathology

**Helene Koefoed-Nielsen ^1,*^, Kristian Kidholm ^1^, Marianne H. Frederiksen ^1,2^ and Minne L.N. Mikkelsen ^1,3,4^**

^1^ CIMT – Centre for Innovative Medical Technology, Odense University Hospital, 5000 Odense, Denmark
^2^ C*I2M - Centre for Integrative Innovation Management, University of Southern Denmark, 5230 Odense, Denmark
^3^ Department of Pathology, Odense University Hospital, 5000 Odense, Denmark
^4^ Department of Pathology, Hospital Sønderjylland, University Hospital of Southern Denmark, Aabenraa, Denmark.
 ^*^ Correspondence: knhelene@gmail.com

Appendix

Tabel 6: Description of the working processes from tissue to diagnosis in a department of pathology before and after the implementation of DIPA. [4]

| **Glass Slide Histology Pathways** | |
| --- | --- |
| **Working flow before and after the implementation of DIPA** | |
| *Before* | *After* |
| ***The laboratory*** | |
| *The reception* | |
| The tissue or organ is received in a container at the department of pathology. The container is equipped with a barcode for further identification. | No change |
| *The grossing* | |
| The organ or tissue is examined by a pathologist or a laboratory technician and areas of interest and areas crucial for further diagnostic are cut out and placed in cassettes with the identification barcode. A macro description is made, and it is described what is in each cassette. | Similar to the workflow before DIPA, but now there is a further special focus on the amount and size of tissue in the cassette. There must not be too much tissue—especially not in the width. |
| *The preparation of tissue* | |
| The cassettes with tissue will be dehydrated in different concentrations of ethanol. | No change |
| *Embedding* | |
| The dehydrated tissue will be embedded in paraffin. Automated embedding will occur as usual prior to DIPA. | Similar to the workflow before DIPA, but now manual embedding will require that only certain cassettes can be used. The large format cassettes should be avoided. |
| *Sectioning* | |
| The paraffin embedded tissue is cut into very thin slices and placed on an objective glass. The automated cutting on the robot will be as prior to DIPA. | The workflow will be as prior to DIPA, but now in manual sectioning, one must be especially attentive to how the tissue is placed on the glass. The tissue must be placed right in the middle, as further lying tissue is at risk of not being scanned due to the scanner profile and its limitations. When one is handling megasections, it is also important that the tissue is not placed in the top of the glass, as the tissue in that situation not will be scanned/understood as tissue but as a barcode. The laboratory will have to standardize the placing of the tissue, as it has to be defined in the software where the barcode is placed and where the scanner will find the tissue. |
| *Staining* | |
| When the tissue is placed on the objective glass it will be stained according to what the pathologist has ordered. If it is special staining or immunohistochemistry, then the stainings will be quality assured on a microscope before the glass can continue the process. | No change |
| *Oven* | |
| **-** | New workstation. When the glass slides are stained, they will be placed in a heating cabinet at 60 degrees for 15 min to avoid the risk that the glass slides will be stuck in the scanner rack, as the coating material sticks to the rack if it is still wet. |
| *Scanning* | |
| **-** | New workstation. All glass slides will now be scanned. The scanners for megasections can scan 30 glass slides at a time or 60 “normal” slides at a time. The ordinary scanners can take up to 360 slides at a time. |
| *Dicomisering* | |
| **-** | New workstation. After the glass slides have been scaned the files or whole slide imaging (WSI) will be sent to a server and converted to the DICOM file format. |
| *Quality assurance 1* | |
| **-** | New workstation. The scanned and converted material (WSI) will be quality controlled. Here the focus is on the scanning, whether the picture of the tissue is clear, if it is possible to see the cells, or if the scanner has focused on dirt or grease on the slide instead of the tissue. |
| *Quality assurance 2* | |
| It is controlled that the tissue agrees with the macro description (measure, right type of tissue, the cutting—not too thick or folds in the tissue). | The principal is still the same as prior to DIPA, but now the quality assurance is made on the WSI. The laboratory technicians working with staining will have to quality assure the staining in the software program and not on a microscope. |
| *Distribution* | |
| The tissue sections will be distributed to the pathologists. The glass is put on trays and put on the respective pathologist’s shelf. | The WSI will be distributed on a computer to the pathologist’s pathology program. In the beginning, the pathologist will receive both the physical slides and the WSI on the computer. |
| ***The pathologists*** | |
| *Receiving the case* | |
| The pathologist can see in a software program that a case is ready. The pathologist has to go and collect the case (slides on a tray) from a specially assigned shelf. | The pathologist receives the case on the computer in a new software program. They click on the case and the WSI opens. |
| *Diagnostics* | |
| The pathologist will take each slide and look at it in the microscope. | The pathologist clicks on each WSI and can also scroll between each WSI. |

Interview guides for qualitative interviews about DIPA.

## **Table 7: Employees, first round.**

| Theme | Main question | Time Estimated |
| --- | --- | --- |
| Intro | About the project, me, and my role  The aim of the interview is primarily to gain insight into   - Your knowledge on DIPA and its implementation - Your views on DIPA - Your expectations for DIPA | 3 minutes |
|  | Ask the person to talk briefly about their educational and work background.  Ask how the person sees himself in terms of technology skills; ask for examples of what other technology the person has (had) contact with? And ask the person to tick off his technology knowledge level on a scale from 1-5. | 5 minutes |
| Theme 1  Views on DIPA  (Strategy) | What do you expect regarding the digitization of the pathology?   - Purpose of DIPA - Pros and cons - Something you see as an disadvantage/obstacle - Something you look forward to | 10 minutes |
| Theme 2  The implementation process.  (style) | Thoughts regarding the implementation process?   - What is your role in the implementation process? - Activities you have participated in or organized? - Has the region and your (other) management been clear about the formulation of your task in connection with the implementation? - Which official success criteria do you work on? - Do you have additional/other success criteria? - Are you in doubt about something? - Is there anything in the first phase of the process (what has been done) that think could have been done differently/had gone differently? - What is the most important thing to focus on in the further implementation process? | 10 minutes |
| Theme 3  Ready for DIPA  (Staff and skills) | Do you feel ready to kick off?   - Are you well equipped (to lead the process)? - Is the department equipped well enough? - Have you and the department achieved competence and suitability in DIPA | 10 minutes |
| Theme 4  Everyday life with DIPA  (Systems, structures) | What do you think about everyday life with DIPA?   - Daily life and activities before DIPA? - What will change after the introduction of DIPA - What does DIPA add? - What limits DIPA - Will DIPA completely replace the existing way of doing things? When? - When is the DIPA project a success? - What milestones (sub-successes) are there along the way? - How do you see yourself in the process – what is your most distinguished role? | 10 minutes |
| Theme 5  Other/additions from the informant  (Shared) | If we must sum it up, what do you think digitization of the pathology?   - How do you feel that DIPA has been received in the department so far? - Differences between employee groups? - What do you think is causing the reception? - Have you thought about how DIPA supports or your values and those of the department? - Will DIPA increase efficiency and collaboration across departments in the region? | 10 minutes |
| Rounding off | Thanks!  A little about the further process. | 2 minutes |

## **Table 8: Employees, second round.**

| Theme | Main question | Time Estimated |
| --- | --- | --- |
| Intro  (Characteristics of the individuals involved) | About the project, me, and my role  The aim of the interview is primarily to gain insight into   - Your views on DIPA - Your perception of the implementation - Your current expectations for DIPA and the further everyday life and commissioning. | 2 minutes |
|  | Technology know-how and technology readiness:  Short summary from last time. Inform what they scored themselves last time. How will they rate themselves in general and how in relation to DIPA.  Has anything in your job function/position changed? If so, when? | 4 minutes |
| Theme 1  Views on DIPA  (Strategy) | What do you think about the digitization of the pathology now?   - The interviewer briefly lists the mentioned advantages and disadvantages from the last interview with the respondent. Hear whether the expectations were right, or something has changed? - Advantages and disadvantages (you lose something or gain something) for you when you work with it. Advantages and disadvantages for others? (Eg colleagues or patients, does the patients get something out of it?) - Something that particularly concerns you after the first months with DIPA? - Have your expectations/assumptions about DIPA been correct? | 15 minutes |
| Theme 2  The implementation process  (style) | How has the implementation process been, seen from your position?   - Activities and training that have taken place have worked well and been relevant to your level. - The doubts that may have existed before commissioning began, have they disappeared? - Is there anything you have seen as either a barrier or a facilitator for a good implementation? - Have there been sufficient resources to support DIPA? - Is it your impression that your colleagues think DIPA is worthwhile? - What do you think about the management of the implementation process? - What is your impression of the super users? Have they been well equipped? - If you were the manager of the implementation process, would you do anything differently? - Something you think would be important to focus on and something that should be done differently | 20 minutes |
| Theme 3  Working with DIPA | Work with DIPA   - Have you acquired the necessary skills and aptitude to work with DIPA? - Are the workstations working? - Are there areas where you need to increase your skills? - Have you been able to get help with problems with the program/equipment? | 15 minutes |
| Theme 4  Everyday life with DIPA | What is everyday life like with DIPA?   - Overall, how has the transition been? - Are you fully digital now? - Did DIPA add anything? For example, has it changed anything in your procedures (good/bad)? - Has DIPA restricted anything? - Are you comfortable working with and using DIPA? (Possibly also ergonomics?) - Has DIPA changed your motivation for work (good/bad)? - Have there been challenges with DIPA? (Possibly ask about a change in response times) - If so, what has been the most difficult part of daily life with DIPA? Could it be approached differently? - Do you experience reactions from the clinics or the other pathology departments? Which ones? - How about quality assurance, is there a procedure for that now - and does it work? - What do you think needs to happen for your everyday life and work processes to function optimally? (Inner and outer setting) - Have you got more or less work? And are you more or less efficient in your flow now? - Are your tasks/functions clear to you? | 10 minutes |
| Theme 5  Motivation for pathology  (Shared values) | If we must sum up, what do you think about digitization of the pathology?   - What is it like to work in pathology today vs. Before DIPA? - Are the things that motivated you still there? - Has DIPA had any impact on what is important to you? - What has DIPA meant for your department/you as a department so far? | 8 minutes |
| Rounding off | Thanks!  A little about the further process. | 1 minute |

## **Table 9: Managers, first round.**

| Theme | Main question | Time Estimated |
| --- | --- | --- |
| Intro | About the project, me, and my role  The aim of the interview is primarily to gain insight into   - Your knowledge of DIPA and the plans regarding its implementation - Your views on DIPA - Your expectations for DIPA | 3 minutes |
|  | Ask the person to tell you briefly about their educational and work background. Where have they been employed and for how long in the current department.  Ask how the person sees himself in terms of technology skills; ask for examples of what other technology the person has (had) contact with? And ask that the person ticks off his technology knowledge level on the scale from 1-5. | 5 minutes |
| Theme 1  Views on DIPA  (Strategy) | What do you think about the digitization of the pathology?   - Purpose of DIPA - Advantages and disadvantages (you lose something, you gain something?) for you when you work with it. Advantages and disadvantages for others. (Eg colleagues or patients, do the patients get something out of it). - Something that worries you - Something you look forward to | 10 minutes |
| Theme 2  The implementation process  (style) | What do you know about the implementation process?   - Activities you have participated in - Has the management been clear about the process, informed about this - Are you in doubt about something? - If you were the manager of the implementation process, would you do anything differently? (not criticizing the current management, but other things you think were important?) (something you think would be important to focus on or something that should be done differently). | 10 minutes |
| Theme 3  Ready for DIPA  (Staff and skills) | Do you feel ready to kick off?   - Are you dressed well enough? - Attainment of competences and suitability (do you have the necessary competences for kick-off during the training course) - What will be most important/what should come first in relation to your education in the implementation process? (where do you need to have more skills?) | 10 minutes |
| Theme 4  Everyday life with DIPA  (Systems, structures) | What do you think about everyday life with DIPA?   - Daily life and activities before DIPA? - What will change after the introduction of DIPA - What does DIPA add? - What limits DIPA - How do you see yourself in this process – are you comfortable in it, can you, for example, keep your motivation up? | 10 minutes |
| Theme 5  Motivation for pathology  (Shared) | If we must sum it up, what do you think digitization of the pathology?   - Why do you work in pathology? - Who are you doing this for? - What motivates you? - What's cool about the job? - Does the introduction of DIPA have any impact on what is important to you? - What will DIPA mean for you as a department? | 10 minutes |
| Rounding off | Thanks!  A little about the further process. | 2 minutes |

## **Table 10: Managers, second round.**

| Theme | Main question | Time Estimated |
| --- | --- | --- |
| Intro  (Characteristics of the individuals involved) | About the project, me, and my role  The aim of the interview is primarily to gain insight into   - Your views on DIPA after it has been in use for some time. - Your perception of the implementation - Your current expectations for DIPA and the further everyday life and commissioning. | 3 minutes |
|  | Technology know-how and technology readiness:  Short summary from last time. Tell what they scored themselves last time and ask where they are now in relation to DIPA. | 5 minutes |
| Theme 1  Views on DIPA  (Strategy) | What do you immediately think about the digitization of pathology now?   - Generally. Has your view of DIPA changed after its implementation?   (Ask about the advantages and disadvantages mentioned in previous interviews)   - Do you think you will achieve what you set out to do with DIPA? | 10 minutes |
| Theme 2  The implementation process  (style) | What thoughts have you made regarding the implementation process?   - How do you feel that the transition from microscope to DIPA has been for the employees. - And how has it been for you (provided the person is, for example, a pathologist)? - What is your impression of the activities and training - have they been comprehensive enough? / Is there something that needs more focus than expected? - How have you experienced leading the department through this implementation process? - Has it required anything in terms of (other than your usual) management style? - Are you in doubt about something currently? - with the region and the other leaders in the pathology departments? - Is there anything in this part of the process (what has been done) that you could have intended to do differently/have gone differently? - Have there been any barriers to implementation? - Any facilitators? - What is the most important thing to focus on in the further process? | 10 minutes |
| Theme 3  Working with DIPA  (Staff and skills) | Work with DIPA   - So far, have you felt well equipped to lead the process? - Do you feel that the department has been prepared well enough to transition to DIPA? - What role have you played so far in the implementation of DIPA? Why? And what do you think that has meant? - Have you and the department achieved the necessary skills and suitability in DIPA – or is there still something left to do? - Based on the experience you have now, when do you think you can talk about DIPA becoming routine for the employees? | 10 minutes |
| Theme 4  Everyday life with DIPA | What is everyday life like with DIPA?   - Has the rollout gone according to plan (neither too long nor too fast?) - Have you reached your milestones along the way? - Are you (the department) fully digital now? - Has the specialty/work environment in the pathology departments become more or less attractive after DIPA? - Has there been talk of standardizing the workflow in the four departments? For example, similar colorings? - Have there been problems along the way with the system? - Is the DIPA project a success? - Or what remains for it to be a success? - With the knowledge you have of it, will DIPA now provide a basis for AI and provide more flexibility across the four departments? | 10 minutes |
| Theme 5  Motivation for pathology  (Shared values) | If we must sum it up, what do you think about digitization of pathology?   - How do you feel DIPA has been received in the department so far? - Differences between employee groups? - What do you think is causing the reception? - Have you thought about how DIPA supports your values and those of the department? - Do you have the impression that DIPA has increased efficiency? - Are you sharing photos across departments now? Has collaboration across the departments in the region increased after the introduction of DIPA? | 10 minutes |
| Rounding off | Thanks!  A little about the further process. | 2 minutes |
